# Relationship Between the Pyroptosis Pathway and Epilepsy: A Bioinformatic Analysis

**DOI:** 10.3389/fneur.2021.782739

**Published:** 2022-01-14

**Authors:** Lu Xia, Lu Liu, Qiang Wang, Jing Ding, Xin Wang

**Affiliations:** ^1^Department of Neurology, Zhongshan Hospital, Fudan University, Shanghai, China; ^2^Department of the State Key Laboratory of Medical Neurobiology, The Institutes of Brain Science and the Collaborative Innovation Center for Brain Science, Fudan University, Shanghai, China

**Keywords:** bioinformatic, pyroptosis, epilepsy, GSDMD, seizure

## Abstract

**Purpose:**

This study aimed to analyse the correlation between the pyroptosis pathway and epilepsy using bioinformatics analysis technology. We analyzed the expression of gasdermin D (GSDMD) and gasdermin E (GSDME), the key molecules of pyroptosis, in kainic acid-induced epileptic mice.

**Methods:**

Weighted gene co-expression network analysis (WGCNA) was used to construct a signed co-expression network from expression data to screen gene sets closely related to epilepsy. The correlation between the module and epilepsy was verified through module conservative analysis, gene ontology (GO) annotation analysis, and correlation analysis with known epilepsy genes. We obtained currently recognized pyroptosis-related molecules through literature review, and correlation analysis was used to evaluate their correlation with epilepsy. Differentially expressed gene (DEG) analysis was used to analyse expression changes of pyroptosis-related molecules at the transcriptome level, compared to the sham group. We subsequently established a kainic acid-induced status epilepticus (SE) model in mice and validated the mRNA and protein expression of GSDMD and GSDME, the key molecules of pyroptosis, by quantitative reverse transcription PCR (qRT-PCR) and western blotting (WB).

**Results:**

Using WGCNA, module conservative analysis, and correlation analysis with known epilepsy genes, we screened out a module (a gene set of interest) closely related to epilepsy that was prominently enriched in immune and inflammatory-related biological processes. Correlation analysis results suggest that pyroptosis-related molecules are closely related to this module, but have no obvious correlation with others. DEG analysis of molecules associated with pyroptosis suggests that most of the pyroptosis-related molecules had significantly increased expression after SE, such as IL1b, Casp1, Casp4, Pycard, Gsdmd, Nlrp3, Aim2, Mefv, Tlr2, Tlr3, and Tlr4. qRT-PCR and WB analysis confirmed that the mRNA and protein levels of GSDMD in the mouse hippocampus were significantly upregulated after SE. The mRNA expression of GSDME was not different between the epilepsy group and sham group. However, the WB results showed that the expression of full-length GSDME was decreased and GSDME-N-terminus were significantly increased after SE.

**Conclusions:**

Our study highlights that the pyroptosis pathway may be closely related to epilepsy. GSDMD and GSDME, the key executive molecules of pyroptosis, will help to understand the pathogenesis of epilepsy and aid in discovering new targets for anti-epileptic drug treatments.

## Introduction

Epilepsy is a common neurological disease affecting over 70 million people worldwide (1). The pathogenesis of epilepsy is complex and still unclear, involving many molecules and signaling pathways. A large amount of evidence accumulated over the past decade has shown that neuroinflammation occurs in animal models of epilepsy and in brain tissue from patients with epilepsy (2–5), which may mediate changes in excitability, extracellular matrix, and synaptic structure and function, contributing toward epilepsy (6–8).

Pyroptosis is a newly discovered method of programmed inflammatory cell death. Unlike apoptosis, pyroptosis is directly mediated by a family of pore-forming proteins called gasdermin, the best characterized of which are gasdermin D (GSDMD) and gasdermin E (GSDME), which lead to the rapid destruction of the integrity of the cell membrane and the release of cell contents and inflammatory molecules, resulting in obvious inflammation (9, 10). An increasing number of researchers have found that gasdermin-mediated pyroptosis is involved in the pathogenesis of multiple sclerosis, cerebrovascular disease, encephalitis, dementia, and other central nervous system diseases, and inhibiting pyroptosis can have a neuroprotective effect in animal models of the above diseases (11–16). However, whether gasdermin-mediated pyroptosis is involved in the pathogenesis of epilepsy remains unclear.

With the development of modern detection technology, bioinformatics analysis of genome-wide expression has provided insights into biological pathways at the molecular level. Transcriptome analysis of the hippocampus has been widely used to investigate the pathogenesis of epilepsy. Previous studies have identified a number of potential genes that may be involved in the pathogenesis of epilepsy through bioinformatics (17–21). This approach provides a good method to analyse whether pyroptosis signaling may be involved in the pathogenesis of epilepsy.

Therefore, the purpose of this study was to analyse the relationship between pyroptosis signaling molecules and epilepsy using bioinformatics. The changes in the expression of key molecules involved in pyroptosis were verified by constructing animal models of epilepsy to further elucidate the role of pyroptosis in the pathogenesis of epilepsy and to provide ideas for finding new therapeutic targets for antiepileptic drugs.

## Materials and Methods

### Data Acquisition of Epilepsy

The datasets were obtained by searching the Gene Expression Omnibus (GEO) and Array Express databases. We systematically searched epilepsy-related studies using the terms “epilepsy” or “seizure.” The inclusion criteria were as follows: (1) data uploaded after 2010; (2) the datasets included both the epilepsy group and the sham group; (3) the minimum sample number per group was 5; and (4) the datasets included samples from the latent and/or chronic stages. Datasets were excluded if they contained only acute-stage samples.

### Weighted Gene Coexpression Network Analysis

The WGCNA package in R was used to construct a signed co-expression network from the expression data (22). The few genes with low expression (mean <0.5) were filtered out. WGCNA was performed using gene expression profile data to construct a gene co-expression network based on the relationship between genes. Genes with similar expression patterns were divided into modules. The module eigengene (ME) of each module was calculated to represent the overall level of gene expression within the module and was used to identify modules highly correlated with clinical traits (23). The epilepsy stage was considered as the acute phase within 1 day (0–24 h) after stimulation, the latent phase was 2–14 days, and the chronic phase was considered after 14 days.

Module preservation analysis in the WGCNA package was used to validate the preservation of modules between the two networks. The thresholds proposed by Langfelder et al. (24) were used to evaluate the degree of preservation (24). The following thresholds for Zsummary were used: a Z-score <2 indicates no preservation, 2–10 indicates weak-to-moderate preservation, and >10 indicates strong preservation.

### Correlation Analysis

Correlation analysis was performed using the GeneOverlap R package (25) and was mainly used for the following two parts: (1) correlation analysis between the module and pyroptosis-related molecules and (2) correlation analysis between the module and known epilepsy-related genes. The gene set of pyroptosis molecules comes from molecules widely mentioned and is generally accepted in reviews of pyroptosis (16, 26, 27). The control group was randomly selected using sample R function. MalaCards (https://www.malacards.org/) is an integrated database of human maladies and their annotations. Epilepsy-related known genes were downloaded from the MalaCards database for subsequent correlation analysis.

### Gene Ontology Annotation Analysis

Gene Ontology (GO) annotation analysis was conducted with the “clusterProfiler” R package. Statistical significance was set at *P* < 0.05.

### Differentially Expressed Genes Analysis

Differentially expressed gene (DEG) analysis was performed using the NetworkAnalyst 3.0 tool (https://www.networkanalyst.ca/). FDR <0.05 and |logFC |>1.5 were considered statistically significant.

### Animal Models of Epilepsy

Male adult C57BL/6 mice (22–26 g, 8–10 weeks) supplied by SPF (Beijing) Biotechnology Co., Ltd. were used in this study. The mice were housed in cages at ambient temperature (22–25°C) and maintained under a standard 12/12 h light/dark cycle. All experiments were approved by the Ethics Committee of Zhongshan Hospital of Fudan University (Shanghai, China) (2019-151). The animals were divided into the epilepsy and sham groups. The epilepsy model was constructed by injecting kainic acid (0.4 μg; Sigma, USA) into the hippocampus at the following coordinates: anteroposterior (AP) = −2.7 mm, mediolateral (ML) = −1.8 mm, and dorsoventral (DV) = −1.7 mm. The sham group received an equal amount of isotonic sodium chloride solution. The epileptic and sham animals were sacrificed 7 days after SE.

### RNA Extraction and qRT-PCR

Total RNA was extracted from the ipsilateral hippocampus of the mice using an RNA purification kit (EZBioscience, USA) according to the manufacturer's instructions. cDNA synthesis was performed using PrimeScript RT Master Mix (Takara, Japan). qRT-PCR was performed using SYBR Green Master Mix (Yeasen, China). Target mRNA levels were normalized to GAPDH levels, and relative expression was calculated according to the 2-ΔΔCt method.

### Protein Extraction and Western Blot

Total protein was extracted using a tissue protein extraction reagent (Beyotime Institute of Biotechnology, China) according to the manufacturer's instructions. Proteins (20 μg) were separated on a 12.5% sodium dodecyl sulfate polyacrylamide gel (Epizyme, China) and then transferred to nitrocellulose membranes (Merck Millipore Ltd., Ireland). After blocking with 5% non-fat milk at room temperature for 1 h, the membranes were incubated with rabbit anti-GSDMD and anti-GSDMD-N(1:1,000, ab219800, Abcam), or anti-GSDME and anti-GSDME-N (1:1,000, ab215191, Abcam) primary antibody overnight at 4°C. The membranes were then incubated with anti-rabbit secondary antibodies at room temperature for 1 h. Images of the bands were captured using a Tenon imaging system (Tanon Science and Technology, China) and analyzed using ImageJ software.

### Statistical Analysis

GraphPad Prism 8 software was used for statistical analysis. Data were represented as (X±s). Two independent samples were statistically analyzed by Student's t-test. *P* < 0.05 was considered statistically significant.

## Results

### WGCNA Analysis

**Figure 1:**
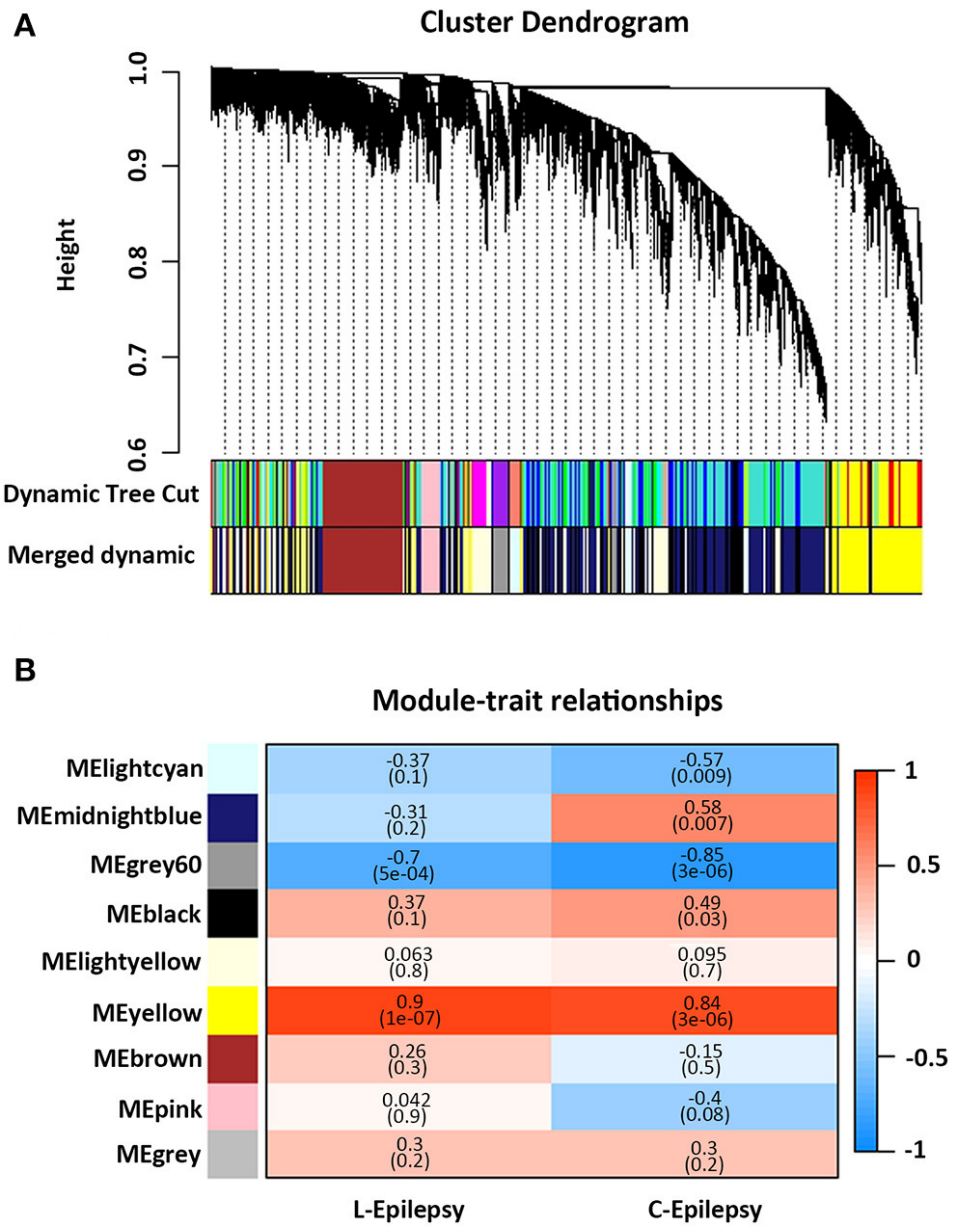
Weighted gene co-expression network analysis. **(A)** Cluster dendrogram of genes based on module eigengenes. The colored bars below cluster dendrogram correspond to different modules. **(B)** Correlation between module eigengene (ME) expression value and epilepsy (sham-−0, epilepsy in latent periods-−1), (sham-−0, epilepsy in chronic periods-−1) for each module. Pearson correlation is reported with the *P* value given inside the bracket.

GSE49849, GSE99577, and E-MTAB-1567 were included in this study, according to the inclusion criteria. The specific information is shown in Table 1. GSE49849 includes both latent and chronic epilepsy samples. During the latent and chronic periods, the pathology of epilepsy changes significantly. Therefore, we selected GSE49849 to construct a weighted gene co-expression network and then performed a conservative analysis with GSE99577 and E-MTAB-1567. A co-expression network was constructed using 18,784 genes from GSE49849, and a total of nine gene modules were obtained after hierarchical clustering and module merging (Figure 1A).

**Table 1:** Characteristics of the datasets in our analysis.

**ID**	**Grouping and number**	**Species**	**Year**	**Data type**
GSE49849	Sham-7d 5; Stimulated-7d 5;	Rat	2013	microarray
	Sham-30d 5; Stimulated-30d 5			
E-MTAB-1567	Sham-5d 5; Stimulated-5d 5;	Rat	2013	microarray
GSE99577	Sham-6h 12; KA-6h 12	Mouse	2019	RNA sequencing
	Sham-1d, 12; KA-1d, 12			
	Sham-2d, 12; KA-2d, 12			
	Sham-4d, 12; KA-4d, 12			
	Sham-6d, 12; KA-6d, 12			
	Sham-12d, 12; KA-12d, 12			

*A total of three datasets were included in this analysis. Seizure was induced by either kainic acid or electrical stimulation in rat or mouse. The type of dataset includes microarray and RNA sequencing*.

As shown in Figure 1B, the yellow module showed a positive correlation with latent and chronic epilepsy (*P* = 1e−07 and 3e−06), while the Gray60 module was negatively correlated with both (*P* = 5e−04 and 3e−06). The Lightcyan module was significantly negatively correlated with epilepsy in the chronic phase (*P* = 0.009). The midnight blue module and the black module were significantly positively correlated with epilepsy in the chronic phase (*P* = 0.007 and *P* = 0.03, respectively) (Figure 1B).

### Module Preservation Analysis

**Figure 2:**
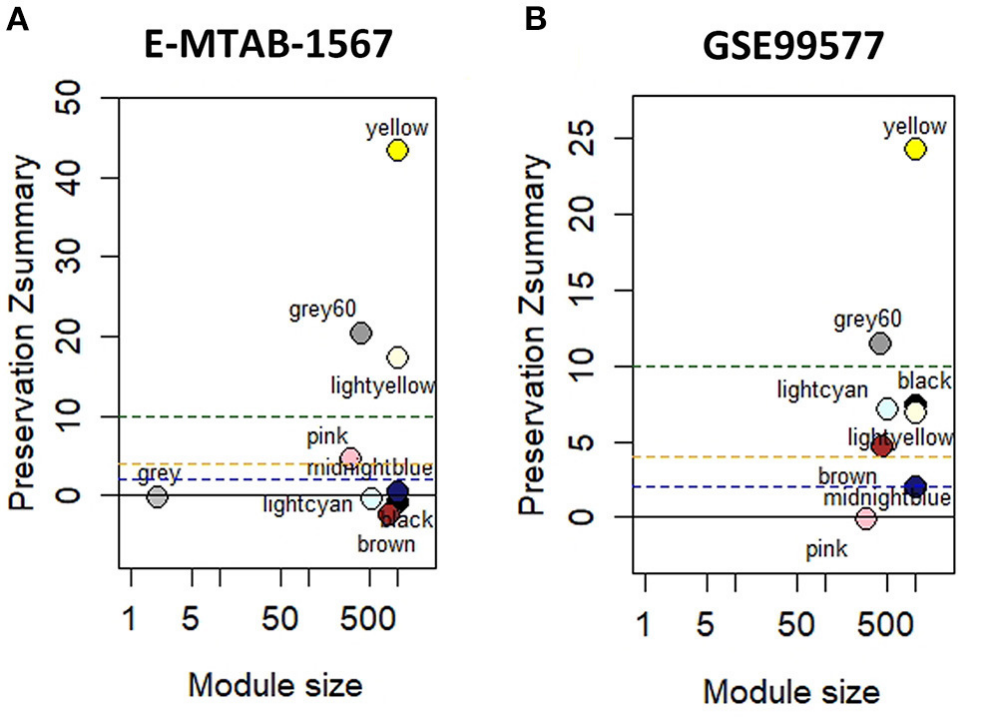
Module preservation analysis with hippocampal datasets. **(A)** Module preservation analysis of GSE49849 vs. E-MTAB-1567. **(B)** Module preservation analysis of GSE49849 vs. GSE99577.

Module preservation analysis was used to identify the modules that were preserved across the conditions. The results of module conservative analysis indicated that the yellow, grey 60, and lightyellow modules were highly conserved (preservation Z-score = 43, 20, and 17, respectively), using E-Mtab-1567 as the test network and GSE49849 as the reference network (Figure 2A). Using GSE99577 as the test network, yellow and grey 60 were highly conserved (Z-score = 24 and 12, respectively) (Figure 2B).

### Correlation Analysis Between the Module and Known Epilepsy-Related Genes

We first obtained known epilepsy-related genes from the MalaCards database, analyzed which modules were significantly related to known epilepsy-related genes, and finally identified key modules with research value. The MalaCards database contains 283 genes associated with epilepsy. After homologous conversion, we obtained a dataset containing 233 genes for subsequent analyses (Supplementary Table 1). Some undetected genes were excluded from the GSE49849 dataset, and 206 genes were included in the correlation analysis. The correlation analysis results indicated that the known epilepsy-related genes were significantly related to the yellow module, but not to other modules (Table 2). Therefore, the results of the analysis suggest that the yellow module is a gene set that is closely related to epilepsy and has significant research value.

**Table 2:** The overlap between modules and known epilepsy-related genes.

**Module**	**Module size**	**Intersection size**	***P*-value**
Black	3,517	34	0.96
Brown	2,213	4	1
Grey	9	0	1
Grey60	571	9	0.28
Lightcyan	830	10	0.58
Midnightblue	5,679	73	0.38
Pink	511	1	1
Yellow	3,086	49	0.038^*^
Lightyellow	2,368	26	0.78

*P-values are obtained using Fisher's exact test by GeneOverlap R package*.

* *P < 0.05. The significant values are shows in bold*.

### GO Analysis

**Figure 3:**
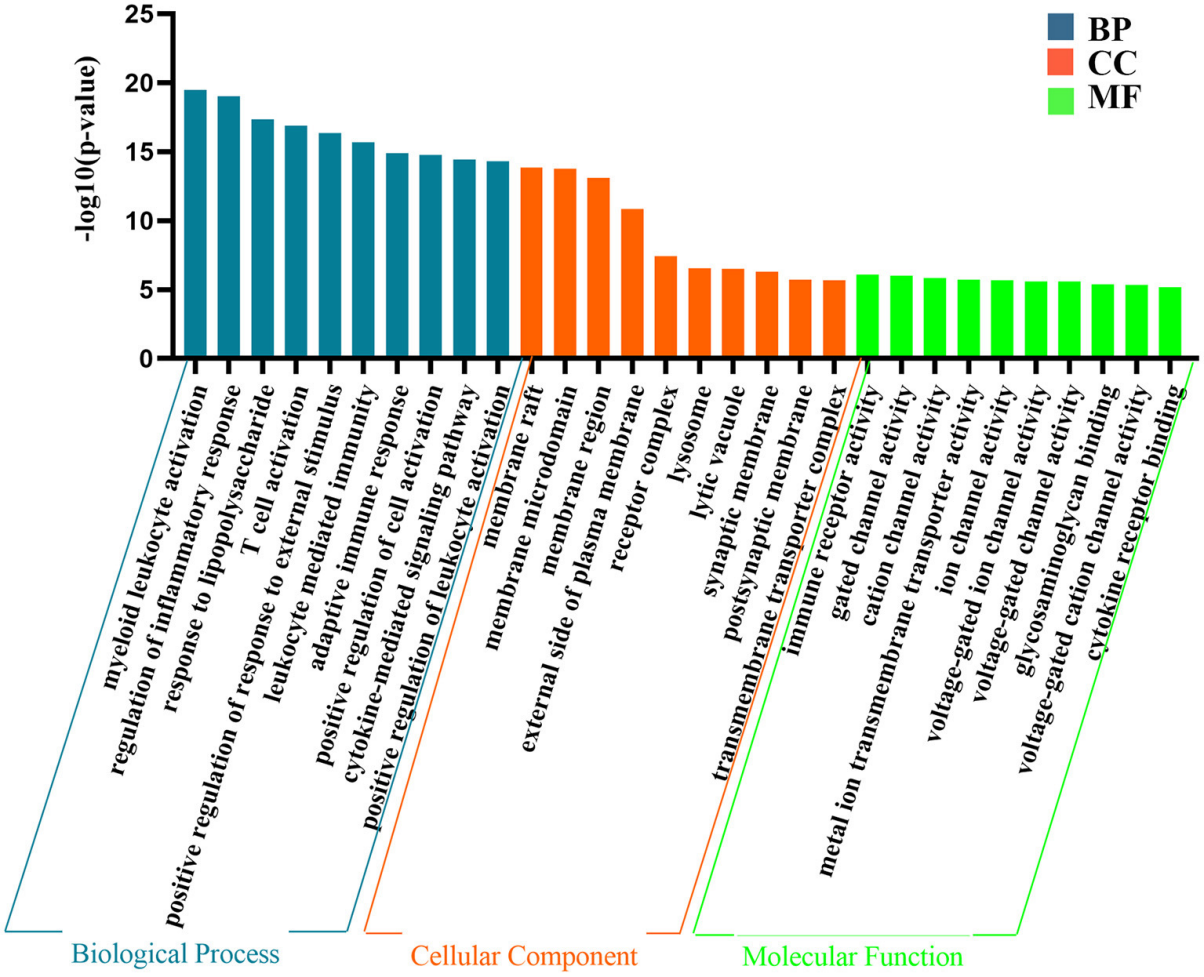
The representative results of GO analysis using yellow module. The top 10 clusters of biological process, cellular component and molecular function were chosen separately according to –log10(*P*-value) ranking and displayed.

The above results indicate that the genes contained in the yellow module are closely related to epilepsy. Next, we performed GO analysis to further analyse their characteristics. As shown in Figure 3, the results of GO analysis suggest that the yellow module was significantly enriched in many immune and inflammatory-related biological processes, such as regulation of the inflammatory response, T cell activation, and positive regulation of response to external stimulus,. Regarding the cellular component, the yellow module was mostly associated with the membrane raft, membrane microdomain, membrane region and lysosome. In terms of the molecular function classification, the yellow module was significantly enriched in immune receptor activity, gated channel activity, and cation channel activity and so on (Figure 3).

### Analysis of the Correlation Between the Module and the Pyroptosis Signaling Pathway

**Figure 4:**
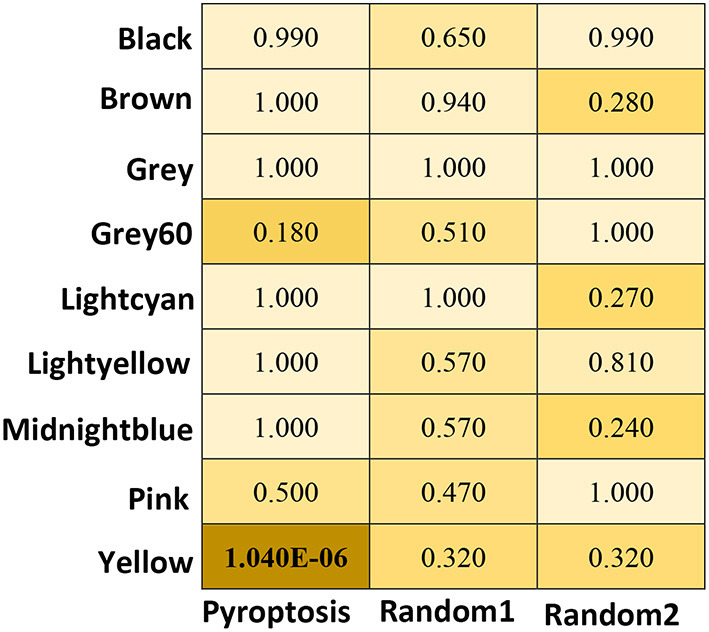
The heat map of correlation Analysis between the module and the pyroptosis signaling pathway. *P* are obtained using Fisher's exact test by GeneOverlap R package. The significant values are shows in bold.

To analyse the relationship between the pyroptosis pathway and epilepsy, we analyzed the correlation between pyroptosis pathway molecules and modules. Twenty-five genes were confirmed to be closely related to the pyroptosis signaling pathway by reviewing studies on pyroptosis (Supplementary Table 2). The analysis results showed that pyroptosis-related molecules were significantly correlated with the yellow module (*P* = 1.04E−06), but with no obvious relationship to other modules. The control group randomly selected by R showed no significant correlation with any of the modules (Figure 4).

### DEG Analysis of Molecules Associated With Pyroptosis

**Figure 5:**
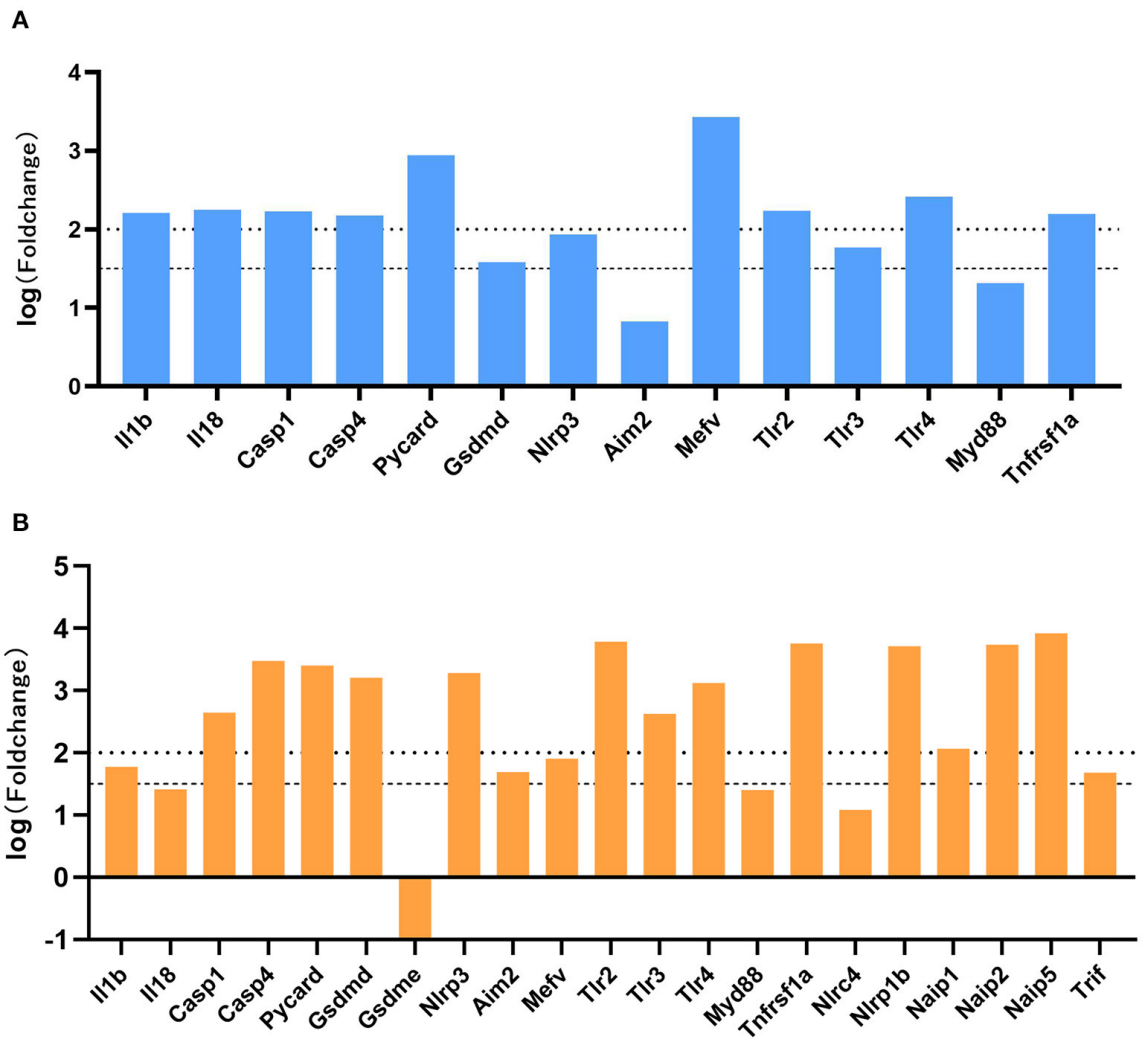
Expression profiles of genes associated with pyroptosis-related molecules. **(A)** The log (fold change) of pyroptosis-related molecules in E-MTAB-1567 is shown. **(B)** The log (fold change) of pyroptosis-related molecules in GSE99577. Fold changes were calculated with respect to the expression of the sham group. A |log (fold change) | >1.5 were considered statistically significant.

We performed differential gene analysis on the two datasets, E-MTAB-1567 and GSE99577. Except for a small number of undetected molecules, all molecules in the pyroptosis signaling pathway meet FDR <0.05. Fold changes (FC) were calculated with respect to the expression of the sham group. A |log (fold change) | >1.5 were considered statistically significant. As shown in Figure 5A, in the E-MTAB-1567 data, the pyroptosis-related molecules with significantly increased expression after SE included IL1b, Il18, Casp1, Casp4, Pycard, Gsdmd, Nlrp3, Mefv, Tlr2, Tlr3, Tlr4 and Tnfrsf1a (Figure 5A). As shown in Figure 5B, in the GSE99577 data, most of the pyroptosis-related molecules had significantly increased expression after SE, such as IL1b, Casp1, Casp4, Pycard, Gsdmd, Nlrp3, Aim2, Mefv, Tlr2, Tlr3, Tlr4, Tnfrsf1a, Nlrp1b, Naip1, Naip1, Naip5 and Trif. Gsdme was the only molecule whose expression level has a slight downward trend after SE, although the difference was not statistically significant (|logFC| <1.5) (Figure 5B).

### The Expression of GSDMD and GSDME in Epilepsy Mice

**Figure 6:**
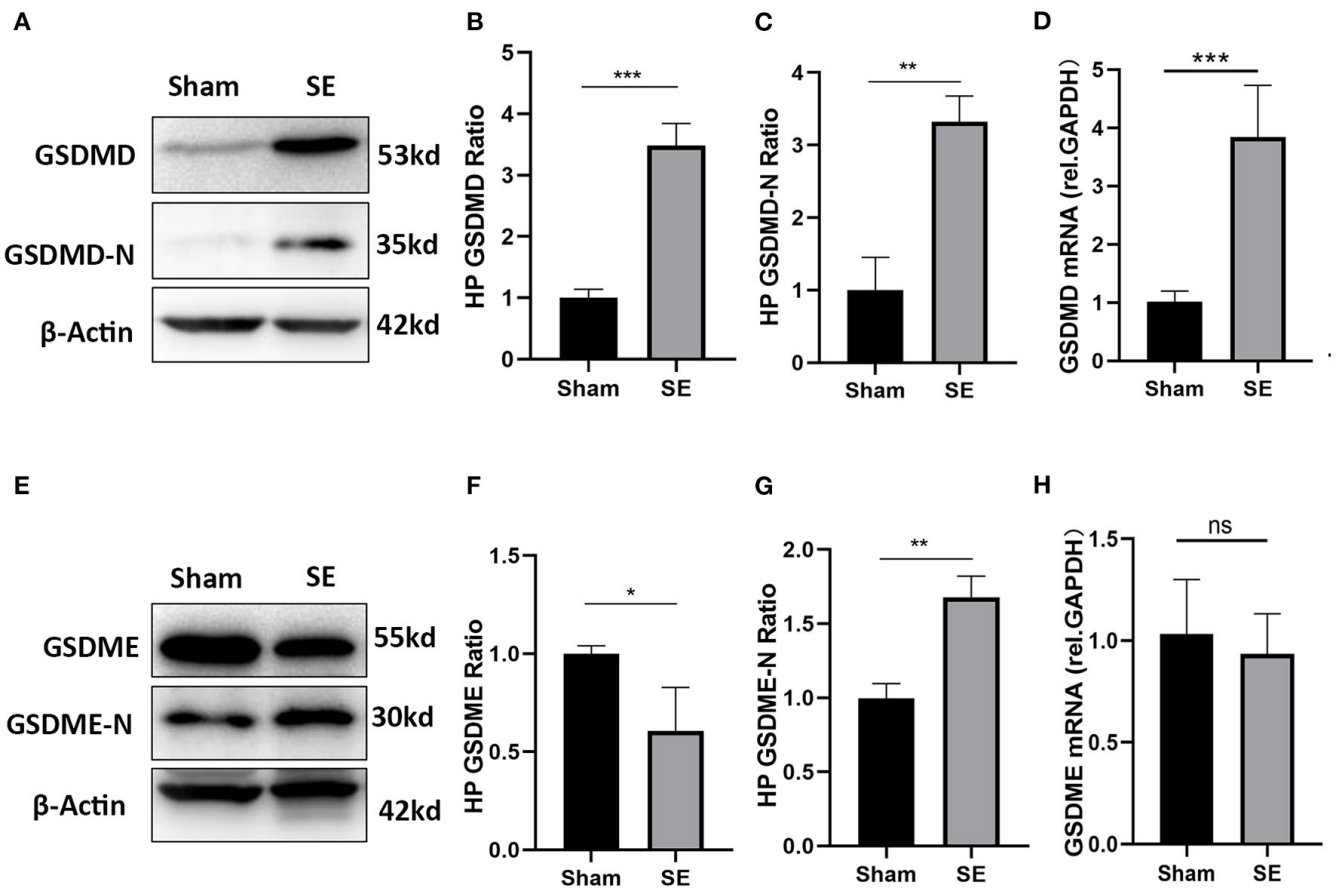
The expression of GSDMD and GSDME in epileptic mice induced by kainic acid. **(A)** WB bands of GSDMD, GSDMD-N and β-actin proteins in the hippocampus. **(B)** Statistical analysis results of the hippocampal GSDMD protein expression in the epilepsy group and sham group. **(C)** Statistical analysis results of the hippocampal GSDMD-N protein expression in the epilepsy group and sham group. **(D)** Statistical analysis results of the hippocampal GSDMD mRNA expression in the epilepsy group and sham group. **(E)** WB bands of GSDME, GSDME-N and β-actin proteins in the hippocampus. **(F)** Statistical analysis results of the hippocampal GSDME protein expression in the epilepsy group and sham group. **(G)** Statistical analysis results of the hippocampal GSDME-N protein expression in the epilepsy group and sham group. **(H)** Statistical analysis results of the hippocampal GSDME mRNA expression in the epilepsy group and sham group (*n* = 3 in each group, * *P* < 0.05, ** *P* < 0.01, and *** *P* < 0.001; HP, hippocampus).

GSDMD and GSDME are key executive molecules in pyroptosis. We established a mouse model of kainic acid-induced epilepsy to verify the expression of GSDMD and GSDME at the transcriptome and protein levels. The qRT-PCR results showed that the mRNA expression of GSDMD was significantly higher in the epilepsy group than in the sham **g**roup (Figure 6D). As shown in Figures 6A–C, both the full-length GSDMD and GSDMD-N-terminus with pore-forming activity were significantly increased after SE (Figures 6A–C). The mRNA expression of GSDME was not different between the epilepsy group and sham group (Figure 6H). However, the western blotting results showed that the expression of full-length GSDME was decreased and GSDME-N-terminus were significantly increased after SE (Figures 6E–G).

## Discussion

Through WGCNA, modular conservative analysis, and correlation analysis, we found a gene set that is closely related to the pathogenesis of epilepsy. GO analysis suggests that this gene set is closely related to neuroinflammation, which is consistent with previous studies (28, 29). Through correlation analysis, we confirmed that the pyroptosis signaling pathway is highly correlated with this gene set. In addition, we found that the mRNA expression of most molecules in the pyroptosis signaling pathway was significantly increased in the epilepsy data. Finally, by establishing an animal model of epilepsy, we verified that the expression of GSDMD-N-terminus and GSDME-N-terminus were significantly increased after epilepsy.

Pyroptosis is an inflammatory type of regulated cell death (RCD) that can be divided into classical and non-classical signaling pathways (30). In classical signaling pathways, pathogen-associated molecular patterns (PAMPs) or damage-associated molecular patterns (DAMPs) stimulate the assembly of a multiprotein complex called the inflammasome, which includes NLRP3, NLRP1, NLRC4, and AIM2. The inflammasome regulates the activation of caspase-1, which promotes the cleavage of GSDMD, and the gasdermin-N domain of GSDMD subsequently forms membrane pores to drive pyroptosis. In non-classical signaling pathways, the GSDMD protein is mainly cleaved by activated Caspase-4/5/11, which drives the occurrence of pyroptosis (16). In addition, the GSDME-N terminal also has the activity to form membrane pores, which is a newly identified mediator of pyroptosis (31). In previous studies, some of these molecules were confirmed to be closely related to epilepsy. High mobility group box 1 protein (HMGB1), a common DAMP pattern, was found to be progressively increased in the nucleus and perinucleus in astrocytes between 1 and 3 h after the onset of kainic acid-induced seizures (32). Moreover, activation of the HMGB1-TLR4-RAGE axis was demonstrated in brain tissue resected during surgery in patients (33). Meng et al. found that the levels of NLRP3 inflammasome components dramatically increased after amygdala kindling-induced SE. Knockdown of NLRP3 or caspase-1 decreased the levels of IL-1β and IL-18 after SE and significantly suppressed the frequency and severity of SRS during the chronic epileptic phase (34). NLRP1 and caspase-1 levels in the resected hippocampus of patients with intractable mesial temporal lobe epilepsy (mTLE) were upregulated (35). However, it is worth noting that GSDMD and GSDME, as the executioner of pyroptosis, has not been studied in epilepsy thus far. In this study, we first verified the abnormally high expression of GSDMD in epileptic mice based on the results of bioinformatics analysis. At the same time, we established a mouse model of kainic acid-induced epilepsy to further confirm that the expression of GSDMD mRNA and protein after epilepsy was significantly higher than that of the sham group. The results of bioinformatics analysis suggest that the expression of most molecules in the pyroptosis signaling pathway were increased after SE. However, GSDME was the only molecule whose expression has a slight downward trend after SE, although the difference was not statistically significant (|logFC| <1.5). Consistent with the results of bioinformatics analysis, qRT-PCR results also confirmed that the mRNA expression of GSDME was not different between the epilepsy group and sham group. However, the WB results showed that the full-length GSDME was decreased and GSDME-N-terminus were significantly increased after SE. We speculate that the transcription level of GSDME is not significantly affected after SE, However, the expression of GSDME-N-terminus cleaved from the full-length GSDME were increased, which played important role in inducing pyroptosis after SE. The specific mechanism of gadermin-mediated pyroptosis after SE deserves further studied in the future. Our results show that GSDMD and GSDME has potential research value in future epilepsy research and provides new ideas for finding anti-epileptic treatment targets. In our study, we found that GSDMD mRNA and protein expression was significantly upregulated after epilepsy, suggesting that GSDMD transcription is increased. We plan to further explore the specific mechanism of its transcription increase in future studies. In addition, there is currently no specific inhibitor of GSDMD and GSDME available, and drug research on GSDMD and GSDME inhibitors will also become one of the major research points.

This study has some limitations. Firstly, the results obtained by bioinformatics analysis are only simple to verify in experimental studies. Further study of other molecules in the pyroptosis pathway should be performed to confirm our conclusions. Secondly, due to the strict inclusion criteria and limited number of available datasets of epilepsy in the GEO database, we only analyzed three sets of data including kainic acid and electrical stimulation-induced epilepsy animal models. Whether the research conclusions are effective in other animal models need further study. Thirdly, due to the lack of available data of epilepsy patients, it is not sure whether our conclusion is applicable to patients with epilepsy.

In summary, our bioinformatics analysis and laboratory verification results suggest that the pyroptosis pathway is closely related to epilepsy. As the key executive molecule of pyroptosis, GSDMD and GSDME may become important breakthrough point, which are expected to provide effective direction for new targets for antiepileptic drug therapy.

## Data Availability Statement

Publicly available datasets were analyzed in this study. This data can be found here: National Center for Biotechnology (NCBI) Gene Expression Omnibus (GEO), https://www.ncbi.nlm.nih.gov/geo/ (GSE49849 and GSE99577). The European Molecular Biology Laboratory's European Bioinformatics Institute (EBMLEBI) ArrayExpress, https://www.ebi.ac.uk/arrayexpress/experiments/E-MTAB-1567/.

## Ethics Statement

The animal study was reviewed and approved by Zhongshan Hospital, Fudan University.

## Author Contributions

LX participated in the experimental performance, bioinformatics analysis, and drafting of the manuscript. LL provided help with image production. QW participated in the manuscript preparation. JD and XW participated in the research design and revised the manuscript. The authors read and approved the final manuscript.

## Funding

This work was supported by project grants from the National Natural Science Foundation of China (Code: 31771184, 81771308, and 31970901) and Shanghai Pujiang Program (code: 19PJ1402200).

## Conflict of Interest

The authors declare that the research was conducted in the absence of any commercial or financial relationships that could be construed as a potential conflict of interest.

## Publisher's Note

All claims expressed in this article are solely those of the authors and do not necessarily represent those of their affiliated organizations, or those of the publisher, the editors and the reviewers. Any product that may be evaluated in this article, or claim that may be made by its manufacturer, is not guaranteed or endorsed by the publisher.

## Abbreviations

GSDMD: gasdermin D
GSDME: gasdermin E
SE: status epilepticus
HP: hippocampus
WGCNA: weighted gene co-expression network analysis
GO: gene ontology
DEG: differentially expressed gene
GEO: Gene Expression Omnibus
PAMPs: pathogen-associated molecular patterns
DAMPs: damage-associated molecular patterns.
