# Supplementary material for: Relationship Between the Pyroptosis Pathway and Epilepsy: A Bioinformatic Analysis
Source: Front Neurol. 2022 Jan 14;12:782739. doi: 10.3389/fneur.2021.782739 (PMC8795950; doi:10.3389/fneur.2021.782739)
Supplement: Supplementary file 4 [file Table_4.docx]

**Figure 6A**

1. （B）


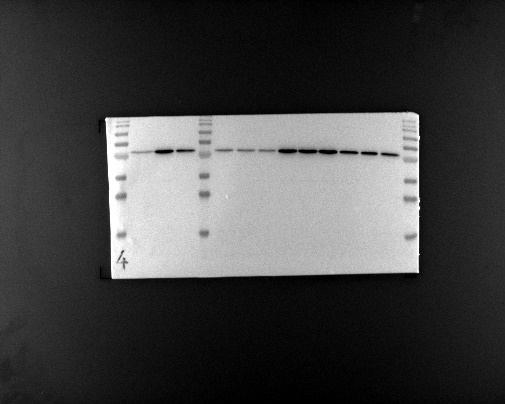

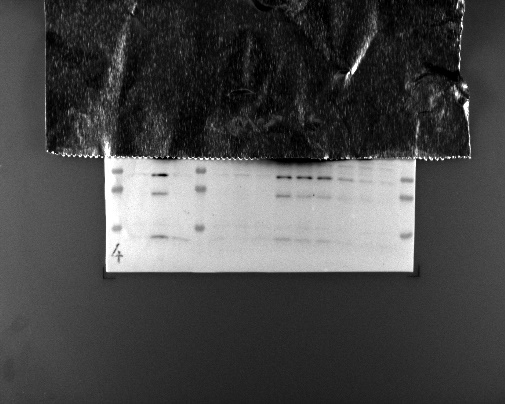


(C)


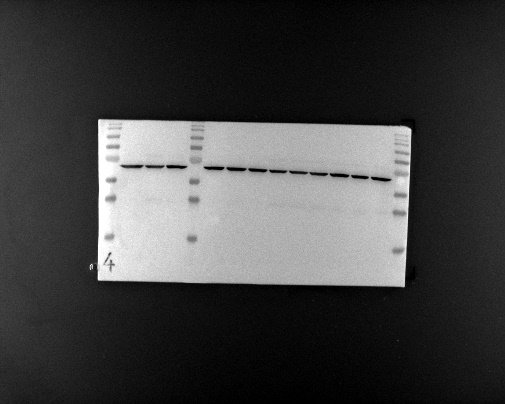


(A) The unedited blot demonstrated the full-length GSDMD in figure 6A.

(B) The unedited blot demonstrated the GSDMD-N in figure 6A.

(C) The membrane of (A) and (B) further incubated with the β-actin antibody. The unedited blot demonstrated the β-Actin in figure 6A

**Figure 6E**

（A） （B）


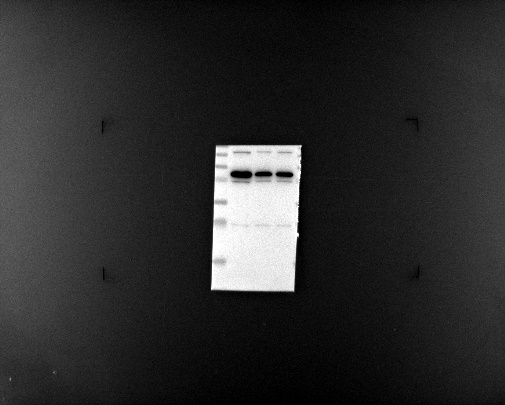

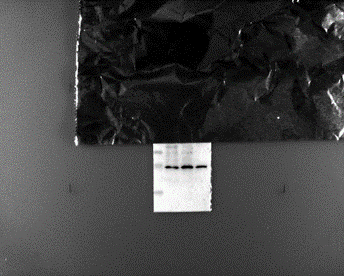


(C)


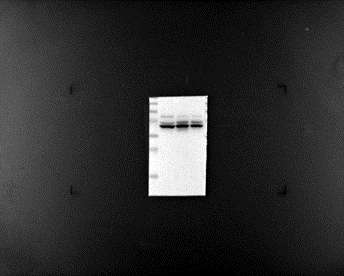


(A) The unedited blot demonstrated the full-length GSDME in figure 6E.

(B) The unedited blot demonstrated the GSDME-N in figure 6E.

(C) The membrane of (A) and (B) further incubated with the β-actin antibody. The unedited blot demonstrated the β-Actin in figure 6E
